# Autopsy Revealed an Extremely Rare Case of Hodgkin Lymphoma With Intranodal Extramedullary Hematopoiesis and Hemophagocytosis in a Patient With Severe Thrombocytopenia

**DOI:** 10.7759/cureus.79298

**Published:** 2025-02-19

**Authors:** Aoi Utsunomiya, Yotaro Asano, Shiori Meguro, Yasuyuki Nagata, Kotaro Nakano, Hideya Kawasaki, Isao Kosugi, Yasunori Enomoto, Satoshi Baba, Toshihide Iwashita

**Affiliations:** 1 Department of Regenerative and Infectious Pathology, Hamamatsu University School of Medicine, Hamamatsu, JPN; 2 Department of Hematology, Hamamatsu University School of Medicine, Hamamatsu, JPN; 3 Department of Hematology, Iwata City Hospital, Iwata, JPN; 4 Department of Preeminent Bioimaging Research, NanoSuit Research Laboratory, Hamamatsu University School of Medicine, Hamamatsu, JPN; 5 Department of Diagnostic Pathology, Hamamatsu University Hospital, Hamamatsu, JPN

**Keywords:** extramedullary hematopoiesis, hemophagocytosis, hodgkin lymphoma, immune thrombocytopenic purpura, lymphadenopathy

## Abstract

In this report, we present an extremely rare case of Hodgkin lymphoma with severe thrombocytopenia, where extramedullary hematopoiesis (EMH) and hemophagocytosis were simultaneously observed within the lymph nodes. Approximately 40 days before the patient’s death, computed tomography performed at another hospital revealed enlargement of deep lymph nodes, including hilar, mediastinal, and mesenteric lymph nodes. Thirteen days before death, the patient was admitted to the hospital with marked hematuria, systemic purpura, mild renal dysfunction, and severe thrombocytopenia; two days later, he was transferred to our institution. On admission, almost all lymph nodes were enlarged, which led us to suspect malignant lymphoma. Blood examination findings suggested that immune thrombocytopenia was a possible cause of severe thrombocytopenia. However, due to severe thrombocytopenia, we could not perform a pathological diagnosis via excisional biopsy of the lymph nodes. Despite treatment with dexamethasone (DEX) pulse therapy, immunoglobulin therapy, and platelet transfusion, the platelet count did not increase. Ultimately, the patient died due to multi-organ hemorrhage. Postmortem pathological examination revealed classic Hodgkin lymphoma in the enlarged lymph nodes, along with EMH and hemophagocytosis within the same lymph nodes. We conducted a literature review and found no prior reports of cases where malignant lymphoma coexisted with EMH and hemophagocytosis within the same lymph node, making this the first reported case.

## Introduction

Autoimmune disorders are frequently reported in association with malignant lymphomas. Immune thrombocytopenic purpura (ITP) associated with malignant lymphoma is a type of secondary ITP and occurs in 0.2-1% of Hodgkin lymphoma cases, with most cases of ITP developing after diagnosing Hodgkin lymphoma [1-3]. Excluding cases of chronic lymphocytic leukemia, ITP reportedly occurs in 0.76% of non-Hodgkin lymphoma cases. In contrast to Hodgkin lymphoma, ITP tends to precede the development of non-Hodgkin lymphoma [4]. The mechanism underlying the association between malignant lymphomas and ITP remains unclear; however, the resolution of ITP following successful lymphoma treatment suggests that lymphoma cells directly or indirectly play a role in the production of platelet-targeting antibodies.

In healthy adults, hematopoiesis occurs exclusively in the bone marrow. However, under certain pathological conditions, such as myelofibrosis, leukemia, or severe anemia (e.g., thalassemia or hemolytic anemia), hematopoiesis can also occur in extramedullary organs, including the spleen, liver, and lymph nodes. According to a case series that described 309 individuals with extramedullary hematopoiesis (EMH) from the Mayo Clinic, EMH was observed in 17 cases of ITP and 24 of malignant lymphoma (including 5 and 19 cases of Hodgkin and non-Hodgkin lymphoma, respectively) [5]. Moreover, one and two cases of Hodgkin lymphoma and ITP, respectively, exhibited EMH within the lymph nodes. These findings suggest that the occurrence of EMH within lymph nodes in patients with ITP or malignant lymphoma is exceedingly rare but possible.

Infectious diseases, autoimmune disorders, and malignancies are commonly associated with hypercytokinemia, which frequently leads to hemophagocytosis by activated macrophages in the bone marrow, spleen, and lymph nodes. Hemophagocytic lymphohistiocytosis (HLH), also known as macrophage activation syndrome, is a disease characterized by high fever, hemophagocytosis, cytopenia, coagulopathy, and liver dysfunction, which can be fatal. Approximately 1% of patients with malignant hematologic disorders reportedly develop HLH [6]. The proportion of cases where hemophagocytosis is observed in lymph nodes among HLH cases associated with malignant hematologic disorders remains unknown.

In this report, we present a case wherein ITP developed before diagnosing the cause of lymphadenopathy. The ITP was refractory to first-line treatment, and the patient ultimately died due to multi-organ hemorrhage. The autopsy revealed the diagnosis of classic Hodgkin lymphoma, with the enlarged lymph nodes showing not only Hodgkin lymphoma but also EMH and hemophagocytosis. To the best of our knowledge, this is the first reported case of malignant lymphoma simultaneously accompanied by both EMH and hemophagocytosis within the same lymph node.

## Case presentation

A 68-year-old Japanese man was admitted to our university-affiliated hospital for further investigation of sudden onset severe thrombocytopenia, lymphadenopathy, anemia, and renal dysfunction. His medical history included diabetes mellitus, dyslipidemia, and bronchial asthma, and he had been prescribed metformin, anagliptin, pitavastatin, levocetirizine, and epinastine.

Here is the course of events leading up to the patient’s admission to our hospital. A non-contrast chest computed tomography (CT) scan was performed at the previous hospital approximately 40 days before death to investigate the ground-glass opacity detected in the right lower lobe on the chest X-ray. The scan revealed infiltration in the right lower lobe, as well as enlargement of the hilar, mediastinal, and mesenteric lymph nodes. Based on these findings, the physicians suspected primary lung cancer or malignant lymphoma. However, due to limitations at their facility, they could not perform a lung biopsy or an excisional biopsy of the enlarged lymph nodes. Therefore, the patient was followed up in the outpatient clinic of that hospital until they could be admitted to our hospital. Twenty-two days before death, the patient had no apparent symptoms, and a blood examination showed no anemia or thrombocytopenia (hemoglobin (Hb): 14.8 g/dL; platelet count: 247 × 10⁴/μL). However, subsequently, the patient gradually developed generalized purpura, gross hematuria, and melena. Due to these symptoms, the patient was admitted to the previous hospital 13 days before death. A blood test revealed anemia or thrombocytopenia (Hb: 9.8 g/dL; platelet count: 0.2 × 10⁴/μL). The patient was subsequently transferred to and admitted to our hospital 11 days before death.

Next, the course of events following the patient’s admission to our hospital is described below. The patient presented to our hospital with palpable lymph nodes in the cervical, axillary, and inguinal regions, each measuring approximately 1-2 cm in diameter. The patient also exhibited systemic purpura and oral bleeding. A blood test revealed that alanine aminotransferase and aspartate aminotransferase were within the normal range; however, albumin was 2.7 g/dL (normal range: 3.8-5.2 g/dL), total bilirubin was 2.1 mg/dL (normal range: 0.3-1.3 mg/dL), and lactate dehydrogenase (LDH) was 446 U/L (normal range: 115-208 U/L). Fibrinogen was normal, prothrombin time (PT) was 15.6 s (normal range: 10-13 s), activated partial thromboplastin time and PT-international normalized ratio were within the normal range, D-dimer was 4.5 μg/mL (normal range: ≤1.0 μg/mL), and anti-thrombin was 60%. These findings met the diagnostic criteria for disseminated intravascular coagulation (DIC). However, DIC alone is unlikely to cause a decrease in platelet count to as low as 0.2 × 10⁴/μL, suggesting the involvement of other factors. Furthermore, considering that the haptoglobin levels were below the detection limit and total bilirubin and LDH were elevated, these findings suggested that, in addition to hemorrhagic anemia due to gastrointestinal bleeding and hematuria, the patient also had concomitant hemolytic anemia.

**Figure 1:**
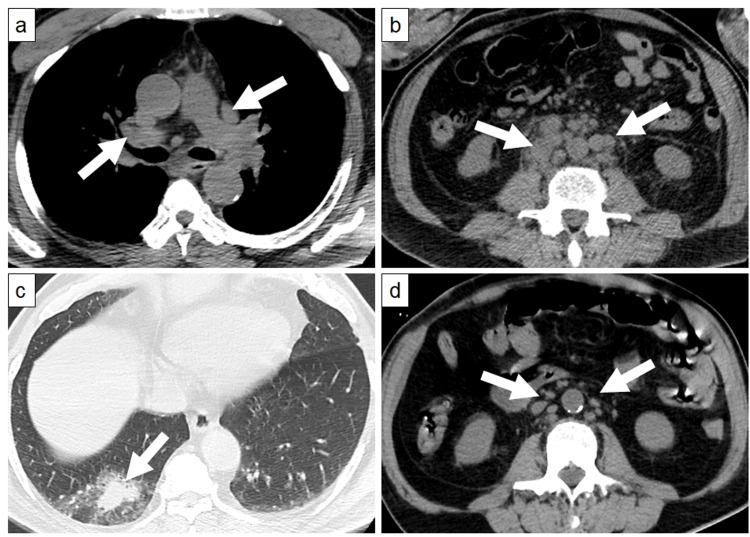
Non-contrasted CT images at hospital admission and on the day of death (a) A non-contrasted CT at admission shows enlargement of hilar and mediastinal lymph nodes (arrows), (b) enlargement of para-aortic lymph nodes (arrows), and (c) consolidation in the right lower lobe (arrow). (d) On the day of death, a non-contrasted CT reveals a reduction in the size of the para-aortic lymph nodes (arrows) compared to the CT at admission (b).

A non-contrast CT performed during admission revealed lymphadenopathy in the hilar, mediastinal (Figure 1a), and mesenteric regions, as well as in the abdominal para-aortic (Figure 1b), cervical, axillary, and pelvic areas. Mild splenomegaly was also observed. The serum levels of soluble interleukin (IL)-2 receptor were markedly elevated (7,269 U/mL). Based on these findings, we suspected malignant lymphoma. Additionally, we suspected the lesion in the right lower lobe to be either a pulmonary involvement of malignant lymphoma or primary lung cancer (Figure 1c). Serum levels of tumor markers, including carcinoembryonic antigen, squamous cell carcinoma antigen, and pro-gastrin-releasing peptide, were all within normal ranges.

**Figure 2:**
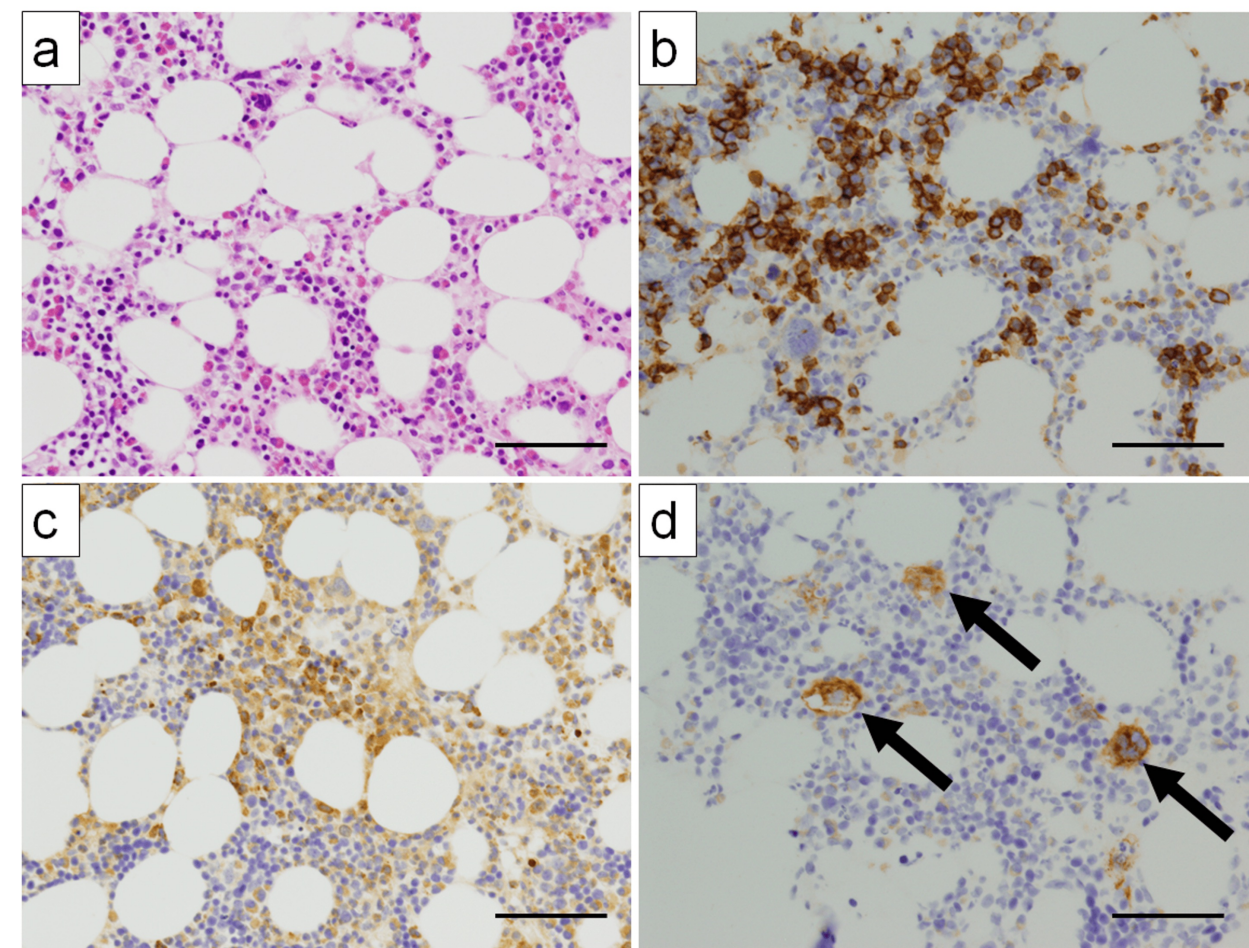
Bone marrow biopsy after admission (a) H&E-stained section of the bone marrow (×200). (b) CD71-positive erythroblastic islands are observed (×200). (c) MPO-positive granulocytic cells are observed (×200). (d) CD41-positive megakaryocytes are observed (×200) (arrows). No apparent morphological abnormalities are observed in erythroid, granulocytic, or megakaryocytic cells. Scale bars: 40 µm. H&E, hematoxylin and eosin

Due to severe bleeding tendency (platelet count: 0.1 × 10⁴/μL, D-dimer: 4.5 μg/mL), biopsies of the lymph nodes and the right lower lung lobe were not performed. Bone marrow aspiration revealed a cellularity of approximately 50% (Figure 2a). The myeloid-to-erythroid (M/E) ratio was within the normal range, with distinct erythroid colony formation and normal myeloid maturation; however, eosinophil levels increased. The number of megakaryocytes was within the normal range. No morphological abnormalities were observed in the erythroblasts (Figure 2b), granulocytes (Figure 2c), or megakaryocytes (Figure 2d). Additionally, we did not observe any blast cells, cancer cells, leukemia cells, malignant lymphoma cells, or hemophagocytosis.

We performed a differential diagnosis for thrombocytopenia. We excluded thrombotic microangiopathy (including thrombotic thrombocytopenic purpura) as we did not observe any schistocytes in the peripheral blood, and von Willebrand factor-cleaving protease (ADAMTS13) activity was within the normal range. We ruled out leukemia and malignant tumor infiltration based on histological examination of the bone marrow. A negative direct Coombs test largely allowed us to exclude Evans syndrome, and the absence of characteristic clinical symptoms enabled us to exclude paroxysmal nocturnal hemoglobinuria as well. Furthermore, tests for antiplatelet antibodies, antinuclear antibodies, and antibodies related to antiphospholipid syndrome were all negative. Antibodies associated with hepatitis viruses, human immunodeficiency virus, human T-lymphotropic virus type I, and Epstein-Barr virus infections were also negative. However, platelet-associated immunoglobulin G was significantly elevated (1,380 ng/10⁷ cells). Based on the above findings, ITP was suspected in the patient through an exclusion diagnosis.

**Figure 3:**
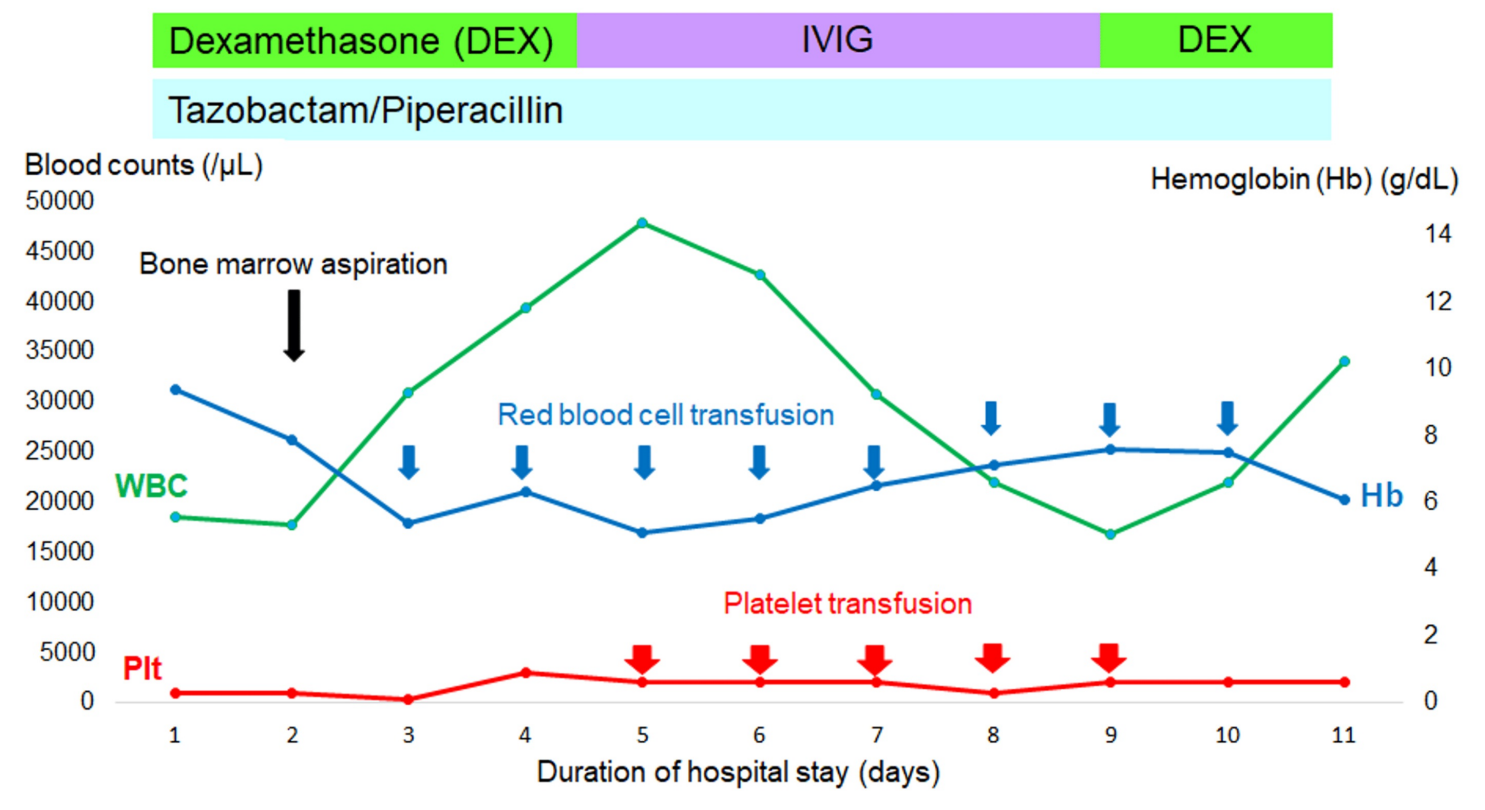
Summary of the clinical course after admission to our hospital On the 5th day of admission, it was confirmed that ADAMTS13 activity was within the normal range, excluding thrombotic thrombocytopenic purpura as the cause of thrombocytopenia. Platelet transfusion was initiated; however, platelet counts remained within the range of 1,000–2,000. IVIG refers to intravenous immunoglobulin therapy.

We planned to address the ITP first, with a lymph node biopsy to be performed once the platelet count reached a safer level. DEX pulse therapy at 0.5 mg/kg/day was administered for four days, followed by intravenous immunoglobulin (IVIG) therapy at 30 g/day for five days (Figure 3). Subsequently, we administered an additional two days of DEX pulse therapy. Daily platelet transfusions of 10 units were administered for five consecutive days. Despite these interventions, the platelet count improved mildly (0.2×10⁴/μL on the day before death), and gross hematuria and hematochezia persisted throughout the 11-day hospitalization. As DEX pulse therapy and IVIG therapy proved ineffective, we planned to initiate rituximab therapy.

Immediately before initiating rituximab therapy, the patient had right flank and right upper abdominal pain. The non-contrast CT performed on the day of death revealed noticeably reduced size of lymph nodes in the pulmonary hilum, mediastinum, abdomen near the aorta, and neck, as well as in the size of the spleen compared with those performed at admission, which we considered to be an effect of DEX pulse therapy (Figure 1d). However, the CT revealed no abnormalities that could explain the right flank pain or right upper abdominal pain. Shortly after the CT scan, the patient lost consciousness, progressed to cardiac arrest, and died. No resuscitation was performed during cardiac arrest.

**Figure 4:**
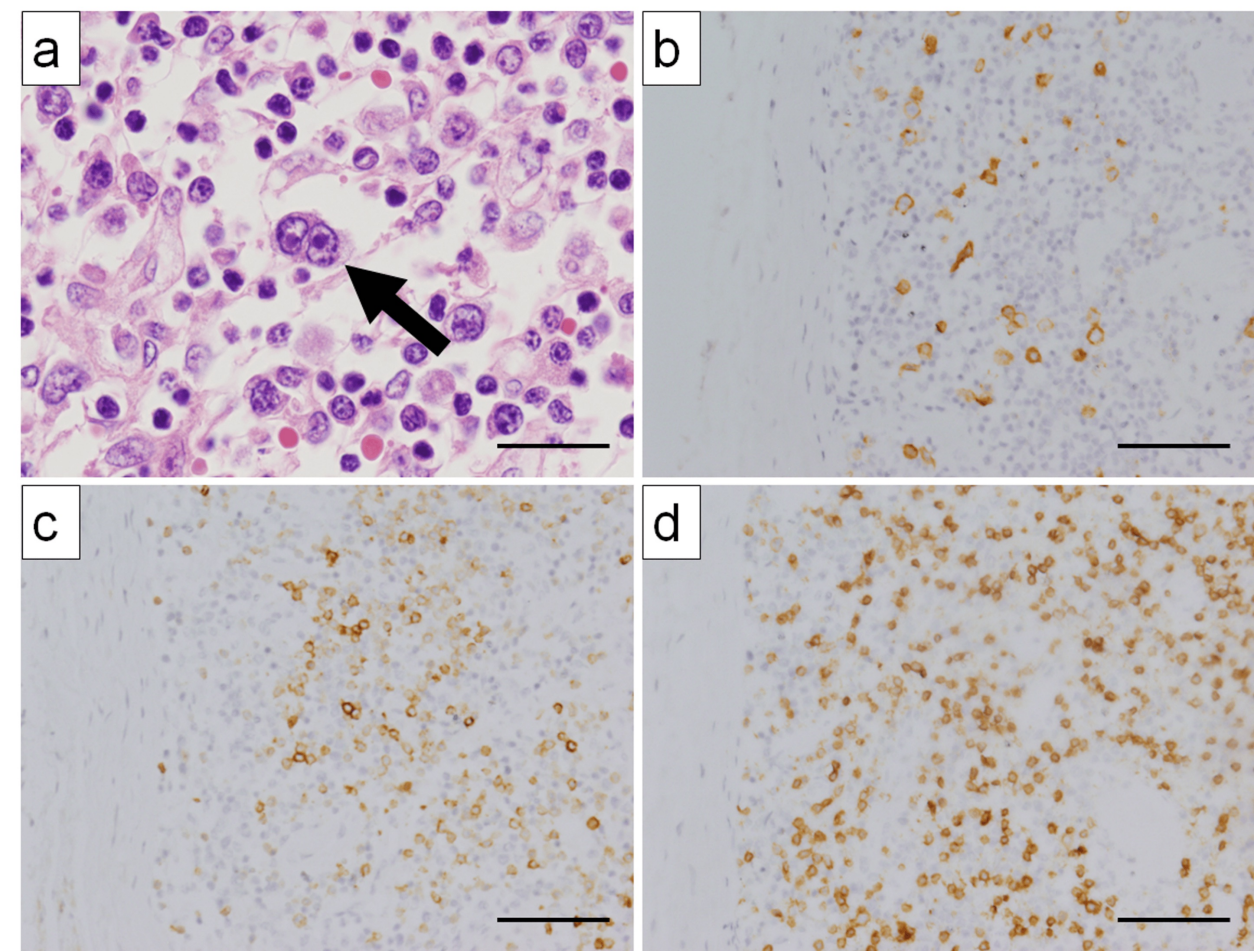
Histological findings of Hodgkin lymphoma in lymph nodes (a) H&E staining reveals a large atypical Reed–Sternberg-like cell with two nuclei (arrow) (×400). (b) Scattered large atypical cells are positive for CD30 (×200). (c) The B-cell marker CD79a is negative in large atypical cells but positive in small normal B cells (×200). (d) T-cell marker CD3 is negative in large atypical cells but positive in small normal T cells (×200). Scale bars: 20 µm (a); 40 µm (b, c, and d). H&E, hematoxylin and eosin

An autopsy was performed to investigate the cause of lymph node swelling. Lymphadenopathy was observed in the cervical, inguinal, hilar, and para-aortic lymph nodes. Microscopic examination revealed the complete loss of normal lymph node architecture in all affected lymph nodes. The large cells were Hodgkin-like and binucleated Reed-Sternberg-like cells with distinct nucleoli (Figure 4a), which were CD30-positive (Figure 4b), CD79a-negative (Figure 4c), and CD3-negative (Figure 4d). In this case, CD30-positive large cells were observed, a finding that can be found in both diffuse large B-cell lymphoma (DLBCL) and angioimmunoblastic T-cell lymphoma (AILT). However, these cells were negative for the B-cell marker CD79a and the T-cell marker CD3, enabling us to exclude DLBCL and AILT, respectively. Ultimately, based on the morphological features in hematoxylin and eosin staining and immunohistochemical findings, a diagnosis of classic Hodgkin lymphoma was made. Epstein-Barr virus-encoded small RNA 1 was negative.

**Figure 5:**
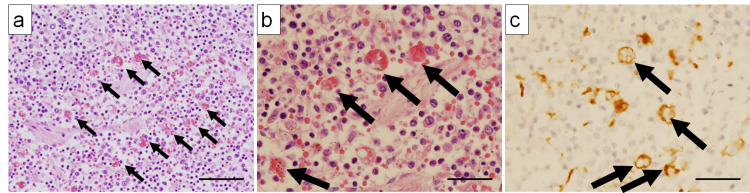
Histological findings of hemophagocytosis in lymph nodes (a) H&E-stained section showing numerous macrophages performing hemophagocytosis within the lymph node (×200) (arrows). (b) H&E-stained section showing macrophages phagocytizing red blood cells (arrows) (×400) (arrows). (c) The cells phagocytosing erythrocytes are CD68 (PGM1)-positive macrophages (arrows) (×400). Scale bars: 40 µm (a); 20 µm (b, c). H&E, hematoxylin and eosin

**Figure 6:**
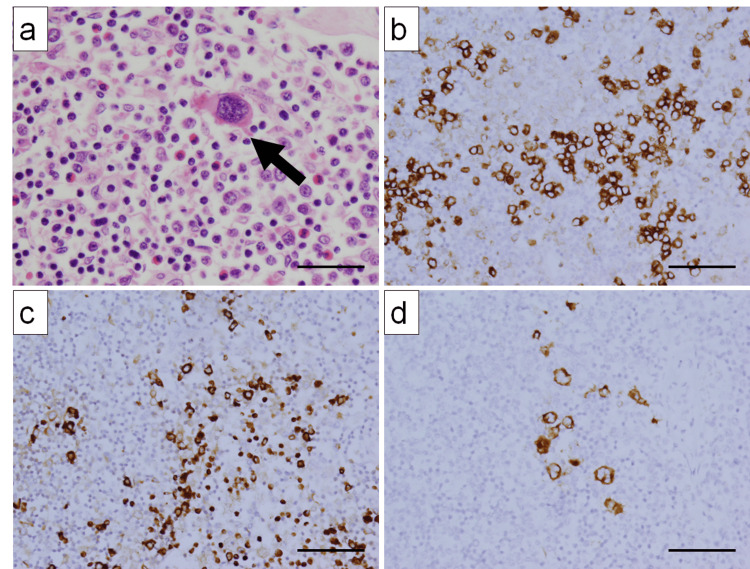
Histological findings of EMH in lymph nodes (a): H&E staining. The large multinucleated giant cell is a megakaryocyte (arrow) (×400). (b) CD71-positive erythroblastic islands are observed (×200). (c) MPO-positive granulocytic cells are observed (×200). (d) CD41-positive megakaryocytes are observed (×200). Scale bars: 20 µm (a); 40 µm (b, c, and d). H&E, hematoxylin and eosin; EMH, extramedullary hematopoiesis

In the cervical and inguinal lymph nodes, hemophagocytosis (Figures 5a-5b) by CD68 (PGM1)-positive macrophages (Figure 5c)) and EMH (including granulocytes, erythroblastas, and megakaryocytes) (Figures 6a-6d) were observed in addition to Hodgkin lymphoma. Macrophages predominantly engulfed erythrocytes. Hodgkin lymphoma cells were also present in the hilar and para-aortic lymph nodes; however, EMH and erythrophagocytosis were less prominent in these sites.

The spleen weighed 140 g and exhibited mild Hodgkin lymphoma infiltration, mild EMH, and mild erythrophagocytosis. Bone marrow aspiration revealed a cellularity of approximately 60-70%. The M/E ratio was within the normal range, with distinct erythroid colony formation and normal myeloid maturation. While eosinophilia was observed in the bone marrow during admission, no increase in eosinophil levels was noted during the autopsy. The number of megakaryocytes was within the normal range. No morphological abnormalities were observed in the erythroblasts, granulocytes, or megakaryocytes. Additionally, the bone marrow showed no evidence of cancer cells, leukemic cells, Hodgkin lymphoma cells, or hemophagocytosis.

The brain weighed 1,240 g, and hemorrhages were identified in the white matter of the left temporal lobe (7 mm in diameter) and around the left hippocampus (2 mm in diameter). No brain herniation was observed. Diffuse hemorrhage was observed throughout the esophagus, stomach, small intestine, and large intestine. Both renal pelves exhibited mucosal hemorrhage; however, no bleeding was noted in the renal parenchyma. The right lower lung lobe exhibited a relatively extensive area of hemorrhage along with a well-defined white tumor measuring 25 × 13 mm, which was histologically diagnosed as mucoepidermoid carcinoma. The heart weighed 430 g, with mild hypertrophy. During the autopsy, minimal pleural and peritoneal effusions were observed. The LDH levels remained stable from admission until death. Additionally, no significant increase was found in uric acid levels, leading us to conclude that tumor lysis syndrome was absent. During death, there was mild hyponatremia (132 mEq/L) and a slight increase in potassium levels (5.2 mEq/L); however, these electrolyte imbalances were not sufficiently severe to induce arrhythmias. The direct cause of death was unclear; however, it was likely attributable to hypovolemic shock caused by severe systemic hemorrhage.

## Discussion

In this report, we presented an extremely rare case of Hodgkin lymphoma with secondary ITP, where EMH and hemophagocytosis were simultaneously observed within the lymph nodes. The patient’s ITP was refractory to treatment and ultimately resulted in death due to multi-organ hemorrhage. Generally, a significant decrease in platelet count makes lymph node excisional biopsy challenging, which can adversely affect the diagnosis and treatment of malignant lymphoma [7]. Although research on the minimum platelet count required for lymph node excision or fine-needle biopsy is limited, the persistently severe thrombocytopenia (0.1×10⁴/μL) present from the time of admission likely precluded the safe performance of an excisional lymph node biopsy in the present case.

Marino et al. reported a case of a 16-year-old man who presented with simultaneous lymphadenopathy and ITP. Due to severe thrombocytopenia (platelet count: 0.1×10⁴/μL), a lymph node excisional biopsy could not initially be performed in this patient; however, following treatment for ITP with DEX therapy and IVIG, the platelet count increased to 3×10⁴/μL, enabling the biopsy, which led to a diagnosis of Hodgkin lymphoma; subsequent lymphoma treatment resulted in near normalization of the platelet count [8].

In this case, similar to that reported by Poponea et al. and Marino et al., the plan was to first address the ITP and allow the platelet count to increase to a safe level for an excisional lymph node biopsy, followed by a pathological diagnosis [7,8]. Unfortunately, the patient’s ITP was refractory to treatment, and the platelet count did not improve. In addition to the findings reported by Poponea et al. and Marino et al., Cecinati et al. also documented a case of ITP associated with Hodgkin lymphoma [9]. However, the reason why ITP is associated with malignant lymphomas, including Hodgkin lymphoma, remains largely unknown. Furthermore, it is entirely unclear why the ITP in the case reported by Poponea et al. and Marino et al. responded to treatment, whereas the ITP in our case did not respond to nearly the same treatment.

According to a case series from the Mayo Clinic, among 309 cases of EMH not associated with myeloproliferative disorders, 19 and 5 involved non-Hodgkin and Hodgkin lymphoma, respectively [5]. Of the five Hodgkin lymphoma cases, EMH in the lymph nodes was observed in one case. Cytokines involved in EMH, such as IL-1α and leukemia inhibitory factor [10], may have been secreted by the Hodgkin lymphoma cells. Interestingly, EMH can also occur in ITP; however, the underlying mechanism remains unclear. Experimental models of ITP in mice have demonstrated an increase in hematopoietic stem cell numbers in the bone marrow, suggesting that such an increase could promote EMH [11].

According to the HLH-2004 guidelines, a diagnosis of HLH requires meeting at least five of the eight diagnostic criteria [12]. In this case, the patient fulfilled only the following four criteria: fever (≥ 38.5 °C), cytopenias, hemophagocytosis in the lymph nodes, and elevated soluble interleukin-2 receptor (7,269 U/mL). Therefore, a diagnosis of HLH could not be established. Schram et al. reported that four of the 68 HLH cases (6%) were associated with Hodgkin lymphoma [13]. In this case, erythrophagocytosis was observed in the lymph nodes and spleen where Hodgkin lymphoma cells were present. However, we observed no erythrophagocytosis in the bone marrow, where Hodgkin lymphoma cells were absent. The serum concentrations of cytokines such as interferon, tumor necrosis factor-α, and IL-6, which activate macrophages, were possibly elevated exclusively in the lymph nodes and spleen containing Hodgkin lymphoma cells.

The lesion in the right lower lobe was identified as mucoepidermoid carcinoma. We observed no evidence of erythrophagocytosis or EMH within or near this malignancy, suggesting no association between mucoepidermoid carcinoma and these phenomena. Additionally, our review of the literature revealed no evidence of a relationship between mucoepidermoid carcinoma and ITP.

The limitation of this case report is that we could not elucidate the molecular mechanisms underlying the reasons for ITP secondary to Hodgkin lymphoma, the treatment resistance of ITP, and the coexistence of EMH and hemophagocytosis with Hodgkin lymphoma.

## Conclusions

We present a case of ITP accompanied by systemic lymphadenopathy. The ITP was refractory to treatment, and despite efforts, bleeding could not be controlled, leading to the patient’s death. The autopsy revealed that the enlarged lymph nodes were due to Hodgkin lymphoma. Notably, within the same lymph nodes, EMH and hemophagocytosis coexisted with Hodgkin lymphoma, a highly rare finding. Considering previous studies indicating that achieving remission of lymphoma can lead to the resolution of ITP, this case underscores the critical importance of prompt lymph node excisional biopsy and pathological diagnosis.
